# Endoscopic intermuscular dissection for management of 10- to 20-mm rectal neuroendocrine tumors: Pilot study (with video)

**DOI:** 10.1055/a-2549-9852

**Published:** 2025-04-04

**Authors:** Silin Huang, Bo Li, Huizhao Deng, Guang Yang, Ronggang Zhang, Jianzhen Ren, Nan Liu, Suhuan Liao

**Affiliations:** 1 Gastroenterology, South China Hospital, Medical School, Shenzhen University, Shenzhen, China; 2 Nephrology and Rheumatology, Shenzhen Hospital of Integrated Traditional Chinese and Western Medicine, Shenzhen, China; 3 Institute of Environment and Health, South China Hospital, Shenzhen University, Shenzhen, China; 4 Marshall Laboratory of Biomedical Engineering, Shenzhen University, Marshall Laboratory of Biomedical Engineering, Shenzhen University, Shenzhen, China

**Keywords:** Endoscopy Lower GI Tract, Endoscopic resection (polypectomy, ESD, EMRc, ...), Tissue diagnosis, Polyps / adenomas / ...

## Abstract

**Background and study aims:**

Endoscopic intermuscular dissection (EID) is associated with higher rates of negative margins in treating rectal neuroendocrine tumors (R-NETs), as reported in case studies. However, evidence regarding the safety and effectiveness of EID remains insufficient. This study aimed to evaluate clinical safety and effectiveness of EID in treating 10- to 20-mm R-NETs.

**Patients and methods:**

Retrospective clinical data from patients with 10- to 20-mm R-NETs who had undergone EID from 2019 to 2024 were collected from a tertiary hospital. The primary outcome was the histological complete resection rate and secondary outcomes included en bloc resection rate and technical success rate.

**Results:**

Twelve patients who had undergone EID were included, with one patient excluded for pathology indicative of a leiomyoma. Among the 11 patients (mean age, 42.45 years; 72.73% males), median diameter was 11.55 mm (interquartile range 10–13 mm). All patients underwent en bloc resection and postoperative pathology confirmed negative horizontal and vertical margins, achieving a histological complete resection rate of 100%. Mean procedure time was 58.55 minutes (standard deviation [SD] 13.66 minutes) and mean postoperative hospital stay was 5.7 days (SD 1.00). One patient developed fever and another experienced abdominal pain, both of which resolved within 24 hours. There were no cases of bleeding or perforation intraoperatively or postoperatively. During a mean follow-up of 31.73 months, there were no residual tumors, local recurrences, or metastases.

**Conclusions:**

EID is a promising treatment for 10- to 20-mm R-NETs, with high initial cure rates, and a new option for endoscopic resection. More studies of the procedure are needed.

## Introduction

Rectal neuroendocrine tumors (R-NETs) exhibit considerable heterogeneity and increased risk of distant and lymph node metastasis as the lesion diameter enlarges 1 . Current guidelines for R-NETs recommend endoscopic excision following detection 2 . Traditional endoscopic mucosal resection (EMR), EMR-related techniques, and endoscopic submucosal dissection (ESD) have been validated as effective therapeutic modalities for R-NETs 3 4 5 6 . However, cases of positive resection margins postoperatively still occur. An innovative technique called endoscopic intermuscular dissection (EID) was introduced previously for treating R-NETs involving the intrinsic muscle layer, with the aim of reducing incidence of positive resection margins 7 . However, there are not yet enough data about the utilization and clinical efficacy of this method. In this article, we present clinical outcomes of patients undergoing EID for R-NETs ≥ 10 mm in a tertiary medical center.

## Patients and methods

This was a retrospective pilot study evaluating efficacy and safety of EID for treatment of R-NETs at a tertiary medical center. Clinical data from patients with R-NETs who had undergone EID from January 2019 to July 2024 were retrospectively collected. The patients met the following criteria: tumors with a diameter ranging from 10 to 20 mm, ultrasound indicating infiltration to the deep submucosal layer, and preoperative enhanced pelvic and abdominal computed tomography (CT)/magnetic resonance imaging (MRI) to rule out distant and regional lymph node metastasis. Patients who underwent EID but whose pathological findings confirmed absence of an R-NET were excluded from the study. These patients were deemed suitable candidates for EID by a multidisciplinary team of experts.

The following data were analyzed: demographic and clinical variables, procedure details, clinical course, and follow-up. The study protocol was approved by the Ethics Committee of South China Hospital of Shenzhen University (HNLS20240412001-A). All patients signed an informed consent form before surgery.

A gastroscope (EG-601WR/EG-580RD, Fujifilm, Tokyo, Japan; HQ260J, Olympus, Tokyo, Japan) was used. An injection therapy needle catheter and Dual knife were used. A transparent distal tapered cap (ST Hood; Fujifilm, Tokyo, Japan) was the preferred distal attachment. Carbon dioxide insufflation and a microprocessor-controlled generator (VIO 200D; ERBE, Tübingen, Germany) were used for all cases. Large vessels were avoided, and major intraprocedure bleeding was treated with hemostatic forceps using soft coagulation; minor bleeding was treated with soft coagulation.

### EID procedure

Endoscopic Intermuscular Dissection: A Surgical Innovation for Rectal Neuroendocrine Tumor Treatment.Video 1

**Fig. 1:**
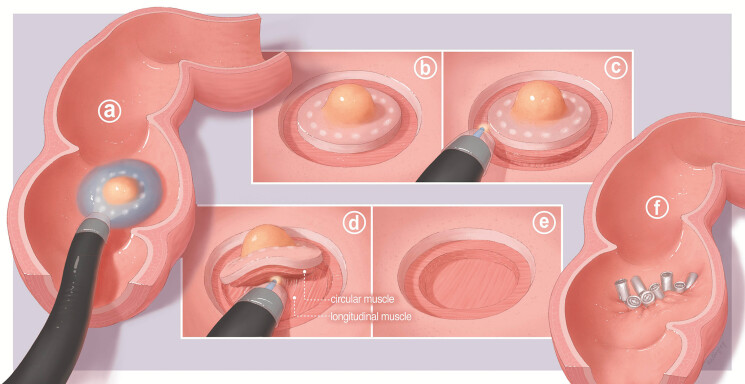
Diagrammatic illustration of EID procedure. **a** SEL lesion was observed in the rectum. Submucosal injection was performed, resulting in good overall lift of the lesion. **b** A circumferential incision was made outside the markers. **c** Circumferential cutting of the muscle fibers from the circular part of the muscle layer, exposing the longitudinal muscle layer. **d** Dissection was performed within the intermuscular space. **e** Postoperative trauma, revealing absence of circular muscle and preservation of longitudinal muscle. **f** Suturing of the defection with metal clips.

**Fig. 2:**
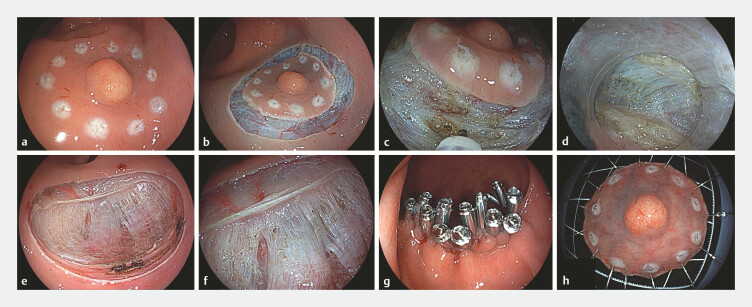
The detailed procedure of EID. **a** A lesion with a yellowish appearance was observed in the rectum, measuring approximately 12 mm in diameter. A circumferential marking was made 5 mm from the edge of the lesion. **b** . A circumferential incision was performed beyond the marked area. **c** Cutting off the circular muscle. **d** Dissection was conducted within the intermuscular space. **e, f** Postoperative trauma, revealing absence of circular muscle and preservation of longitudinal muscle. **g** Suturing of the defection with metal clips. **h** The resected tumor.

All endoscopic procedures were performed under general anesthesia with endotracheal intubation by an expert endoscopist with extensive experience, who had conducted over 100 cases of ESD and 100 cases of peroral endoscopic myotomy (POEM). EID was subsequently conducted in accordance with a structured five-step process ( Fig. 1 , Fig. 2 , Video 1 )

Step 1. Lesion marking and submucosal injection. The periphery of the lesion was marked using a soft coagulation (efficacy 4, power 80 W). Subsequently, multiple injections of physiological normal saline containing indigo carmine were administered around the marked points to create a submucosal cushion.

Step 2. Circumferential incision. A circumferential incision was performed beyond the marked area, followed by dissection of the submucosal layer to expose the muscularis propria (Endo Cut Q, effect 3).

Step 3. Intermuscular dissection. An ST hood was affixed to the endoscope tip, facilitating severing of circular muscle fibers to access the intermuscular space. This step allowed for the exposure of the longitudinal muscle layer, followed by continued dissection within the intermuscular space until complete tumor resection was achieved. (Endo Cut Q, effect 3)

Step 4. Hemostasis and closure of the mucosal incision. Diligent hemostasis was achieved for soft coagulation (efficacy 4, power 80 W). If exposed vessels were present, electrocautery was applied for hemostasis before closure using clips or a combination of clips and nylon sutures.

**Fig. 3:**
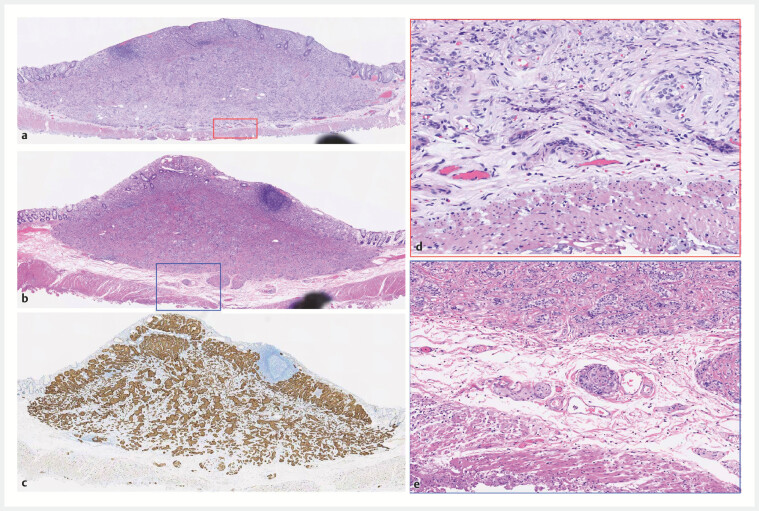
Examples of postoperative pathology indicating R0 resection. First patient: 10-mm tumor. **a** The tumor was closely adjacent to the intrinsic muscle layer, with the resected circular muscle layer visible beneath (×4, H&E). **d** Tumor cells with round nuclei and pale cytoplasm arranged in a ribbon-like pattern, closely abutting the muscularis propria (× 20, H&E). Second patient: 10-mm tumor. **b** The tumor was situated deep within the submucosal layer, with focal clusters of cells closely apposed to the muscularis propria (×4, H&E). **e** The tumor lacked a capsule and the cells displayed a scattered cluster growth pattern, also in close proximity to the muscularis propria (×20, H&E). **c** Synaptophysin (Syn) staining was positive, indicating neuroendocrine differentiation (×4, H&E).

Step 5. The specimens were meticulously stretched and secured on foam boards using entomological pins, followed by immersion in formalin for at least 24 hours prior to routine pathological and immunohistochemical examinations ( Fig. 3 ).

Postoperative pathology was evaluated by a senior pathologist.

### Post-EID management and follow-up

Following surgery, patients abstained from oral intake for 24 hours before resuming a liquid diet. Intravenous prophylactic administration of third-generation cephalosporins was conducted for 3 days, with continuous monitoring of vital signs. Patients were discharged once they had no fever or pain and were able to tolerate a liquid diet.

Surveillance procedures included colonoscopy and thoracoabdominal enhanced CT or MRI at 6 to 12 months postoperatively. If no recurrence or metastasis was detected, subsequent colonoscopies and abdominal CT were performed annually for a consecutive 3-year period.

### Study outcomes and definitions

The primary outcome was histological complete resection (R0) rate, defined as complete en bloc resection of the targeted lesion with both horizontal and vertical free margins. Secondary outcomes included the following: 1) En bloc resection rate, deﬁned as complete single resection of the targeted lesion, irrespective of whether the basal and lateral tumor margins were inﬁltrated or undetermined; 2) Procedure time, measured from initiation of marking to completion of mucosal incision closure; 3) Procedure success rate, deﬁned as the proportion of patients whose tumors were successfully resected; and 4) Complications, including intraoperative and postoperative complications, deﬁned as perforation or hemorrhage occurring during or after the operation. Perforation was deﬁned as an endoscopically visible hole in the rectal wall, postoperative clinical symptoms of peritonitis, and/or radiological evidence of free air under the diaphragm. Bleeding was classiﬁed as either procedural or delayed. Procedural bleeding was deﬁned as arterial bleeding or active oozing for more than 30 seconds during the procedure that necessitated endoscopic, radiological, or surgical intervention. Delayed bleeding was deﬁned as postoperative clinical symptoms, such as hematochezia within 14 days after the procedure, requiring endoscopic, radiological, or surgical intervention. Postoperative complications also included fever and abdominal pain.

Histopathological grade included NET G1 (Grade 1): mitotic count less than 2 per 10 high-power fields (HPF), Ki-67 index less than 3%; NET G2 (Grade 2): mitotic count 2–20 per 10 HPF, Ki-67 index 3%-20%; NET G3 (Grade 3), mitotic count greater than 20 per 10 HPF, Ki-67 index greater than 20%, according to the 2022 WHO classification 8 .

Gastrointestinal and pancreatic neuroendocrine tumors were staged according to the 8th edition of the American Joint Committee on Cancer (AJCC) 9 . Length of hospitalization was deﬁned as the total number of days from hospitalization to discharge. Local recurrence referred to reappearance of tumor cells at the site of the original resection and within 1 cm of its vicinity, manifesting at least 6 months after surgery. Residual tumor referred to presence of tumor remnants detected at the original resection site and within 1 cm of its surroundings within the first following surgery.

### Statistical analysis

SPSS version 27.0(IBM Corp., Armonk, New York, United States) was used to perform statistical analysis. Continuous variables were reported as means with standard deviations (SDs) or medians with interquartile ranges (IQRs), as appropriate. Categorical data were expressed as frequencies (%).

## Results

### Baseline characteristics

Twelve patients who had undergone EID were included, with one patient excluded after postoperative pathology indicated a leiomyoma. Among the 11 patients, the mean age was 42.45 ± 7.6 years and 72.23% were male; all cases presented with solitary lesions. The majority of lesions (8/11, 72.23%) were located in the lower rectum. Maximal tumor diameter ranged from 10 to 15 mm, with a median diameter of 11.55 mm (IQR, 10–13). Eight cases (72.73%) were classified as 0-Is and three cases (27.27%) were classified as 0-IIa according to the Paris classification. The predominant color was yellow in eight cases (72.73%). All patients underwent preoperative endoscopic ultrasound (EUS), which confirmed that all lesions originated from the deep submucosal layer, with a predominant hypoechogenic pattern. Preoperatively, all patients (100%) underwent abdominal enhanced CT to rule out regional and distant metastases, and patients with tumor diameters of 13 mm and 15 mm also underwent preoperative 68GaPET-CT to exclude metastases ( Table 1 ).

**Table:** **Table 1** Baseline characteristics of study patients (n = 11).

Outcomes	Results
Gender, male, n (%)	8 (72.73)
Age, mean (SD), years	42.45(7.6)
Location, n (%)	
Upper	1 (9.09)
Middle	2 (18.18)
Low	8 (72.73)
Diameter, median (IQR), mm	11.55 (10–13)
Preoperative EUS	
Submucosa, n (%)	11(100)
Preoperative MRI or CT scans to rule out metastasis	11 (100)
Paris classification, n (%)	
0-Is	8 (72.73)
0-IIa	3 (27.27)
Color, n(%)	
Yellow	8 (72.73)
White	3 (27.27)
CT computed tomography; EUS, endoscopic ultrasound; IQR, interquartile range; MRI, magnetic resonance imaging; SD, standard deviation.	

### Primary and secondary outcomes

All 11 patients (100%) underwent en bloc resection via EID. Postoperative pathology confirmed grade G1 with negative horizontal and vertical margins, achieving a histological complete resection rate and a technical success rate of 100%. According to the 8th edition of the AJCC staging for gastrointestinal and pancreatic neuroendocrine tumors, a substantial majority of the patients (90.91%; 10 /11) were categorized in stage T1, while a smaller percentage (9.09%; 1/11) were classified in stage T2. Among the 11 patients, median distance from the lower margin of the tumor to the muscularis propria was 232 um (0–747).

Mean procedure time was 58.55 minutes (SD 13.66) and mean postoperative hospital stay was 5.7 days (SD 1.00). One patient experienced low-grade fever postoperatively, which was considered to be a result of postoperative stress response. Prophylactic antibiotic therapy was administered as per routine, and the patient's temperature returned to normal within 24 hours. Another patient experienced mild abdominal pain postoperatively with no signs of perforation, which resolved spontaneously within 24 hours without requiring intervention. There were no instances of intraoperative or postoperative bleeding or perforation complications. Regular follow-up was completed for all 11 patients, with a mean follow-up of 31.73 months (SD 16.97). There were no residual tumors, local recurrences, or metastases ( Table 2 ).

**Table:** **Table 2** Intervention and hospitalization characteristics (n = 11).

Outcomes	Results
Primary outcome	
Histological complete resection (R0) rate, n (%)	11 (100)
Secondary outcomes	
Operation time, mean (SD), minutes	58.55 (13.66)
Success rate of operation, n (%)	11 (100)
En bloc resection rate, n (%)	11 (100)
Hospital stay, mean (SD), days	5.7 (1.00)
Intraoperative complications, n (%)	
Bleeding	0 (0)
Perforation	0 (0)
Postoperative complications, n (%)	
Bleeding	0 (0)
Perforation	0 (0)
Fever	1 (9.09)
Abdominal pain	1 (9.09)
Pathological outcomes	
Grade (G1/G2/G3), n	11/0/0
T1/T2	10/1
The distance from the lower margin of the tumor to the muscularis propria (um)	232 (0–747)
Positive vertical margins, n (%)	0 (0)
Positive horizontal margins, n (%)	0 (0)
Positive vascular invasion, n (%)	0 (0)
Follow-up outcomes	
Follow-up time, mean (SD), months	31.73 (16.97)
Postoperative recurrence, n (%)	0 (0)
Postoperative residue, n (%)	0 (0)
Metastasis, n(%)	0 (0)
SD, standard deviation.	

## Discussion

Rectal NETs are the most common neuroendocrine tumors in the gastrointestinal tract, comprising approximately 48% of gastrointestinal NETs 10 . Their incidence has increased in recent years 11 . With the growing prevalence of colonoscopy, most R-NETs are detected in early stages, typically locally with a relatively low risk of distant metastasis and a relatively high 5-year survival rate 12 . R-NETs exhibit significant heterogeneity, with an increasing risk of distant and lymph node metastasis as lesion size increases. Factors predisposing to R-NET metastasis include tumor size, infiltration of the muscularis propria, pathological classification, vascular invasion, and atypical endoscopic features 2 13 . Tumor size is considered the most significant predictor of R-NET metastatic risk 14 . R-NETs smaller than 10 mm in diameter carry a mere 1% risk of distant metastasis, whereas those larger than 2 cm exhibit a metastasis rate as high as 60% 15 . Accurate preoperative assessment of R-NETs is crucial for selecting surgical approaches and predicting prognosis. Preoperative evaluation of R-NETs primarily involves imaging studies such as EUS, CT, MRI, or positron emission tomography-CT (PET-CT) 16 17 . In this study, 12 patients underwent preoperative EUS examination and enhanced CT scans, which indicated that all tumors were located in the deeper submucosal layer, with no metastases detected in any of the examinations.

Various endoscopic techniques are available for local resection of R-NETs, including traditional EMR, EMR-related techniques, ESD and anal surgery. Currently, no absolute optimal recommendation exists in guidelines for R-NETs for tumors measuring 10 to 20 mm and they should undergo local excision or radical surgery. In addition, small R-NETs are sometimes misdiagnosed as polyps and inadvertently snared, resulting in a high risk of incomplete resection, warranting salvage therapy. Complete resection of R-NETs is paramount, and ensuring adequate vertical margin depth is crucial, emphasizing the imperative for endoscopists to minimize occurrence of postoperative positive margins.

EID is a novel technique initially employed for severe fibrotic rectal lesions and suspected deep submucosal invasion of rectal early carcinoma. We first reported application of the EID technique for treatment of R-NETs 7 , with more reports of EID treatment for related diseases such as residual R-NETs, rectal gastrointestinal stromal tumor, and gastric NET 18 19 20 21 . However, current reports about EID for R-NETs are limited to case reports, necessitating further clinical research to validate procedure safety and efficacy. Here, we report the results of the descriptive pilot study of EID for treatment of R-NETs with a diameter of 10 to 20 mm. Our results indicated that en bloc resection was successfully achieved in all patients, confirming the technical feasibility of this technique. Pathological examination confirmed negative horizontal and vertical margins, indicating an R0 resection rate of 100%, significantly higher than the 82.6% R0 resection rate reported for ESD treatment of R-NETs in previous studies 22 . For R-NETs with a diameter of 10 to 20 mm, endoscopic full-thickness resection (EFTR) is also an option. Current literature reports R0 resection rates for EFTR treatment of rectal subepithelial lesions (SEL) ranging from 87% to 100% 23 24 25 , with inconsistencies across studies. It has also been observed that there are some complications following EFTR surgery. In addition, lack of availability of the full-thickness resection device required for EFTR in some regions also limits the development of this technique.

EID utilizes natural anatomical layers, dissecting under endoscopy between the inner circular muscle and outer longitudinal muscle of the muscularis propria, excising the mucosal layer, submucosal layer, and circular muscle, while preserving the longitudinal muscle and serosal layer without perforation. This approach thereby maintains organ integrity and reduces the rate of positive vertical margins when the deep submucosa is invaded 26 . In cases of postoperative residual, due to the scar, submucosal dissection can be challenging. By shifting the dissection plane to the space between the inner circular muscle and outer longitudinal muscle, dissection difficulty is reduced while ensuring specimen integrity and complete lesion excision 7 .

In our study, this technique demonstrated favorable outcomes, with patients recovering well postoperatively. Only one patient developed a low-grade fever, and another experienced mild abdominal pain, both of which resolved within 24 hours. There were no cases of intraoperative or postoperative bleeding or perforation complications, and no recurrences or residual tumors were observed during an average follow-up period of 31 months, indicating the safety of this technique. Given that our case accumulation is still relatively limited, compared with ESD, EID, which involves resection of the circular muscle, is associated with a relatively higher risk of postoperative infection. Therefore, in management of patients after EID, we remain more cautious. We administer prophylactic third-generation cephalosporins postoperatively to prevent infectious complications. Whether prophylactic antibiotic treatment is unnecessary if the longitudinal muscle layer is not damaged, and the duration of prophylactic antibiotic treatment, are also among the issues that we are highly concerned about and need to investigate further.

Postoperative pathology confirmed that all 11 cases were G1 grade, with 10 cases being stage T1 and one case being stage T2. We observed that among these 11 cases, the major tumors are situated in the deep submucosal layer, whereas some tumor cells exhibit focal or nodular clusters and detach from the primary mass, extending more deeply into the submucosa layer and some closely adjoining the muscularis propria, as illustrated by two cases in Fig. 3. Our data showed that the median distance from the lower margin of the tumor to the muscularis propria was 232 um (0–747). This propensity contributes to the positive margins commonly observed with EMR or ESD. This is why we recommend EID rather than ESD for lesions larger than 1 cm in diameter and located in the deep submucosal layer, as suggested by EUS. Consequently, compared with ESD, EID more effectively ensures negative margins, thereby reducing the need for reoperation.

In our experience, all patients underwent endotracheal intubation anesthesia, with muscle relaxants used during anesthesia to relax rectal muscles and facilitate intermuscular dissection. The primary challenge of this technique is dissection of the muscular layer, which requires precise differentiation between the inner circular muscle and the outer longitudinal muscle. This procedure necessitates that it be performed by highly skilled senior physicians who possess extensive experience in ESD and POEM. Furthermore, strategic employment of underwater and traction techniques during EID can mitigate the complexity of separating the intermuscular spaces and also enable early detection of intermuscular perforating vessels, allowing for proactive management and thereby minimizing risk of intraoperative bleeding 18 27 28 . Indications for EID are currently not clearly defined but our experience suggests that patients should undergo pelvic and abdominal MRI or 68Ga PET-CT scans, as well as EUS, to rule out distant metastasis and lymph node involvement. For lesions smaller than 10 mm or those larger than 10 mm but with a relatively thick submucosal layer as indicated by EUS, ESD treatment is sufficient. However, if EUS suggests that the lesion is located in the deep submucosal layer, we still recommend EID as the more suitable option. This recommendation, however, requires further evidence to support it.

The primary limitation of this study is its retrospective design and the small sample size, which may constrain generalizability of our findings. Future research endeavors should focus on multicenter, prospective studies to further substantiate the indications, effectiveness, and safety of this technique. Despite these limitations, our study stands out as the inaugural investigation to present EID outcomes, providing valuable preliminary data in this area.

## Conclusions

In summary, high rates of R0 resection, along with low complication rates, suggest that EID is a safe and effective treatment option for R-NETs. Its superior initial cure rate underscores its potential as a new endoscopic resection method for managing non-metastatic R-NETs with a diameter of 10 to 20 mm. However, more high-level evidence is needed to further study the procedure.

### Data availability statement

The datasets supporting the conclusions of this article can be made available upon request.
